# The Association between the Police, Ambulance, Clinician Early Response (PACER) Model and Involuntary Detentions of People Living with Mental Illness: A Protocol for a Retrospective Observational Study

**DOI:** 10.3390/nursrep13040122

**Published:** 2023-10-13

**Authors:** Julia Heffernan, Amy Pennay, Elizabeth Hughes, Richard Gray

**Affiliations:** 1 School of Nursing and Midwifery, La Trobe University, Bundoora, Melbourne, VIC 3086, Australia; r.gray@latrobe.edu.au; 2 Centre for Alcohol Policy Research, La Trobe University, Bundoora, Melbourne, VIC 3068, Australia; a.pennay@latrobe.edu.au; 3 School of Health and Social Care, Edinburgh Napier University, Edinburgh EH11 4BN, UK; l.hughes@napier.ac.uk

**Keywords:** PACER, police, ambulance, mental illness, involuntary detention, protocol

## Abstract

Emergency services are frequently called to attend mental health incidents and are looking for innovative ways to improve their responses and reduce the burden on services. Involuntary detention of people living with mental illness is considered more frequent than necessary, leading to increased pressure on emergency departments, and is often a traumatic experience for patients. The Police, Ambulance, Clinician Early Response (PACER) model was developed in 2019 in Canberra, Australia, and seeks to reduce involuntary detentions by embedding a mental health clinician into emergency services as a mobile mental health crisis response intervention. This protocol details a retrospective cohort study that will examine the association between PACER and involuntary detentions using medical and police records and compare the results to standard ambulance and police responses. We will use relative risk and odds ratio calculations to determine the probability of being involuntarily detained or diverted from hospital; and we will describe the patient characteristics and outcomes in the PACER cohort. Results will be reported using the STROBE checklist for reporting cohort studies. This study was not registered on a publicly accessible registry.

## 1. Introduction

### 1.1. Mental Health and Emergency Department Statistics

There is some evidence of an increase in the prevalence of mental illness in Australia. The National Health Survey conducted by the Australian Bureau of Statistics reports that 20% of Australians experienced a mental illness between 2017 and 2018, representing an increase of 3% since 2007 [1,2]. It is estimated that 45% of Australians will experience mental illness in their lifetime [2]. Increased emergency department (ED) presentations and inpatient mental health unit demands are consequences of increased rates of mental illness [3,4,5,6,7]. Mental health services are not emergency services; rather, they provide secondary specialist services across government and non-government sectors. For people experiencing a mental health crisis, particularly those exhibiting suicidality, psychosis, and mania, police and ambulance services are often the first point of contact and the primary responding service [8,9,10,11,12,13,14,15,16].

The prevalence of mental illness is difficult to estimate globally. A systematic review of 174 surveys from 63 countries reported prevalence estimates from 1980 to 2013 [17]. The review and meta-analysis found that approximately 18% of adults identified as meeting criteria for a high-prevalence mental illness during the preceding 12 months, and that the lifetime burden of mental illness was approximately 29% [17]. The authors of the World Mental Health Survey Initiative reported that the highest prevalence of mental illness is in the United States (27%) and the lowest in Nigeria (6%), with anxiety and depressive disorders being the most prevalent [18].

A Global Burden of Disease study estimated that the global prevalence of mental illness had risen from 665 million cases in 1990 to 970 million in 2019 [19].

The COVID-19 pandemic has seen a probable increase in depression and anxiety due to exacerbation of many determinants of poor mental health, with the pandemic estimated to have caused an additional 53 million cases of depressive disorders, and 76 million cases of anxiety disorders globally, representing an increase of 28% and 26%, respectively [20].

The rates of mental health-related ED presentations in Australia have increased by approximately 3% per year since 2016 [3,21]. According to more recent data, 47% of mental health-related presentations were attended by ambulance vehicles, representing twice the utilization of ambulance services for non-mental-health-related presentations [21]. This indicated an increased rate of ambulance presentations of people living with mental illness to the ED from 47% to 52% during the period from 2020 to 2021 [21]. A further 6% of mental health-related presentations were brought in by police or corrective services [21]. Whilst this appears to be a relatively low occurrence, it represents 10 times the rate of non-mental health patients attending the ED via police and corrective services (1%) [21].

The Australian Institute of Health and Welfare reported that only one third of patients attending an ED for a mental health-related presentation went on to be admitted for psychiatric care (39%) [3,21]. The remaining patients were assessed, and their episode of care ended without being admitted to hospital [3,21]. At the study site in the Australian Capital Territory (ACT), the rate of admission following mental health-related ED presentation was 44%, with 51% being discharged after assessment, and the remaining patients failing to wait to be seen [21].

Patients attending the ED for mental health-related presentations tend to wait longer to be seen than patients who present for non-mental health concerns [22]. In the United States, patients waiting to be seen for mental health reasons can wait up to 5–12 h longer than non-mental health patients, which often relates to the need for medical clearance, mental health symptom management, ambulance arrival, and after-hours presentation [22,23].

ED activity data have demonstrated increased mental health-related presentations in the United Kingdom (UK) and United States (US), including significant increases in presentations by children and young people, particularly for self-harm-related symptoms [23,24,25,26].

### 1.2. Mental Health Legislation and Involuntary Detention

Each Australian state and territory has local mental health legislation which contains provisions for the involuntary detention of a person suspected to be living with mental illness, for the purpose of compelling them to an ED or hospital for compulsory mental health assessment. Those afforded these powers are typically doctors, mental health clinicians, police, and ambulance paramedics. In the Australian Capital Territory (ACT), the Mental Health Act 2015, Section 80 contains these involuntary detention provisions [27].

Within the legislation, specific criteria must be met for the person to be involuntarily detained. Typically, the criteria for police and ambulance paramedics are lesser or more generalized than that of doctors and mental health clinicians, as they are not considered experts in identifying mental illness. Police and ambulance paramedics require only a reasonable belief that the person is living with mental illness, and that they pose a risk to themselves or others. However, a mental health clinician or doctor is required to distinguish mental illness with certainty, make a prediction that the person will deteriorate without immediate treatment, and, most notably, that the person is refusing treatment and care due to the symptoms of mental illness.

Involuntary detention provisions provide invasive and restrictive powers that are allowed for the completion of that detention. This includes legal entry into the person’s home using force, such as breaching doors and windows, personal and property searching and removal of items, physical holds and restraint, and forcible giving of medication.

In an Australian qualitative study, 122 patients who had direct contact with a police/clinician co-responder mental health crisis team were followed for 2 weeks after crisis contact [7]. The authors reported that diverting mental health patients from the ED was important in terms of minimizing the potential trauma caused to the patient through involuntary detention, transportation, and ED assessment, as well as reducing the resource impacts on emergency services and EDs [7]. This is consistent with the 5th National Mental Health and Suicide Prevention Plan (2017), whereby patient-centered care, choice, and increased options for community mental health care are an Australian government commitment [28].

Furthermore, mental health legislation in Australia has a strong focus on least restrictive care, which is interpreted as the least intrusive course of action being considered before moving to more restrictive methods, whilst giving patients the right to refuse treatment or engagement with mental health services, where possible [27].

### 1.3. Impact of Involuntary Detention on Patients

A narrative synthesis review of qualitative studies explored patients’ experiences of involuntary detention during an acute period of mental illness [29]. The review included 15 studies and reported several negative themes including loss of perceived independence, feelings of terror, worsening of paranoid beliefs, and increased distress [29]. Interventions involving restraint, forcible giving of medication, and sedation were commonly described as “terrifying” and activated pre-existing vulnerabilities, powerlessness, and re-traumatization of those with previous experience of abuse, and was associated with feelings of being bad and being punished [29,30,31,32,33,34].

Other patients reported that the involuntary detention process triggered anger and rage, with corresponding dramatic escalation of agitation and behavioral disturbance, whereas others described withdrawal associated with feeling disempowered [29,30,31,32,33,34]. Additionally, a UK-based systematic review of 56 qualitative studies relating to patients’ experiences of involuntary detention and assessment found similar themes, with patients reporting fear and distress during the detention, particularly when they experienced use of force and restraint [30].

### 1.4. Impact of Involuntary Detention on Police and Ambulance Services

Despite limited training opportunities to increase mental health literacy, the rate of mental health-related presentations being referred to ambulance services has increased [4,15,35]. A scoping review examined the existing literature relevant to how paramedics respond to people experiencing a mental health crisis [35]. Across 14 included articles, consistent themes were noted, including the absence of mental health training opportunities, concerns about paramedic scope of practice in the management of patients with acute mental illness, organizational policies and procedures, and legislative requirements [35]. Some of the included studies highlighted that paramedics feel an obligation to transport patients living with mental illness to hospital, and not doing so was akin to failing to provide a service or fulfil an obligation [35]. Time constraints were also cited as a rationale for transporting patients to hospital, as was the perception that mental illness was not considered a priority condition by paramedics [35].

An Australian cohort study examined the involuntary detentions by paramedics over a 3-month period, using medical records [36]. The study demonstrated that the rate of hospitalization for involuntarily detained patients was much lower for paramedic detentions (27%) than for those detained by doctors or mental health clinicians (60%) [36]. After adjusting for covariates such as triage category (urgency) and presentation time, the odds of hospitalization after involuntary detention were three times lower in the paramedic cohort than with doctors and authorized clinicians [36]. This suggests that paramedics are more likely to involuntarily detain patients who do not need hospital care.

These themes are consistent with international data which demonstrate a high prevalence of mental health-related presentations to ambulance services. A data linkage study examined the epidemiology of mental health-related ambulance calls in Scotland [4]. The study linked data from the ambulance service, EDs, and inpatient units across a 12-month period and found that 11% of ambulance calls were coded as either mental health or self-harm (*n* = 9014) [4]. Transportation to and discharge from hospital was the primary outcome of ambulance attendance to mental health-coded patients (*n* = 4566, 51%) [4]. Eleven percent of patients (*n* = 1003) were left in the community and the remaining were admitted to a hospital ward (*n* = 2043, 23%). The authors concluded that the rate of transport to an ED and subsequent discharge for people experiencing a mental health problem demonstrated a missed opportunity to improve patient outcomes and reduce the burden on services [4].

Several studies have demonstrated that mental health patients transported to an ED by police are more likely to be discharged home after assessment than those brought in by mental health professionals [37]. One such study examined the outcomes of patients involuntarily detained by police in an Australian state using medical and police records and a 167-patient sample [37]. Of these, 130 were discharged home after assessment (67%), with the remaining experiencing involuntary and voluntary mental health admissions to hospital or medical admission or absconding [37]. The authors concluded that there was an opportunity for police to explore alternative options to involuntary detention that promote less restrictive options, reduce transportation to EDs, and reduce traumatic patient experiences [37].

### 1.5. Rates of Involuntary Detention

There is evidence from observational research of increasing rates of involuntary detentions [3,25,26,38,39,40,41,42]. Involuntary detention rates across 22 states in the US demonstrated an annual increase of 13% for people of all ages between 2012 and 2016 [40].

An Australian study reported similar findings, demonstrating a 262% increase in the use of involuntary detentions by police and ambulance paramedics [7]. The data in this study showed that involuntary detentions by police represented two-thirds of all detentions, however, the rates invoked by ambulance paramedics had more than doubled from 2004 (15%) to 2010 (38%) [7]. Of those involuntarily detained by either police or ambulance, less than half went on to be admitted to hospital for psychiatric care [7].

Whilst involuntary detention will always be appropriate for certain patient presentations, there are several well-documented justifications for reducing the overall numbers including reducing trauma, reducing demand on health and emergency service resources, and consistency with overarching strategic mental health plans and standards. The increasing prevalence of mental illness, corresponding ED presentations, and increased rates of involuntary detentions add further resourcing and financial burdens on health and emergency services with negative impacts on hospital wait times and patient experience. With this in mind, alternative options to divert patients from hospital into community mental health services may be an appropriate and efficient intervention.

### 1.6. Involuntary Detention Provisions in the ACT

The ACT has in place the Mental Health Act (2015) which gives involuntary detention powers to doctors, mental health clinicians, police officers, and ambulance paramedics [27].

In the act, police and ambulance paramedics have very general criteria to meet, whereas doctors and MHOs have stricter, more extensive criteria, given that they are expected to make a more informed clinical assessment of the patient.

This difference in criteria is associated with greater numbers of patients being involuntarily detained by police and ambulance paramedics than with doctors and clinicians. Furthermore, those detained by doctors and clinicians are significantly more likely to be admitted to a mental health inpatient unit. This can be interpreted as doctor and clinician application of involuntary detention provisions being more clinically informed than those invoked by police or ambulance.

Due to the stark differences in criteria under the act, the ACT has observed a significant increase in patients being placed under involuntary detention by emergency services, with approximately 86% being assessed as not requiring hospitalization, and subsequently discharged. The rate of patients being brought into an ED under involuntary detention by the ambulance service is approximately four times higher than the number of patients brought into an ED by clinicians. Police also bring in a high number of patients subject to involuntary detention who go on to be released immediately after assessment. For the purpose of this research, these are considered “unnecessary” in that the person was involuntarily detained, assessed, and released as requiring immediate treatment and care for their mental illness, as defined in the act.

### 1.7. ACT PACER Model

The Police, Ambulance, Clinician Early Response (PACER) model differs from usual service provisions as a tri-response mobile emergency mental health service that provides a mental health clinician, police officer, and ambulance paramedic who attend mental health crises in the community together in a first responder vehicle. PACER can act as either a primary or secondary police/ambulance response and has access to mental health background information and the three agencies’ electronic records. From the perspective of the patient, PACER offers early intervention to a mental health crisis, potential diversion from hospital ED, avoidance of potential delays, and may reduce involuntary detentions, use of force, and associated trauma.

The police officer has the primary role of maintaining the safety of the patient and the PACER team, the paramedic has the role of providing medical care including medical clearance, wound care, and emergency care; and the clinician has the responsibility for the patient’s mental health assessment and involuntary detention decision making.

### 1.8. Other PACER Models

Few tri-response PACER models are in operation around the world. Co-response models are being utilized more frequently, which pairs a police officer or a paramedic with a mental health clinician to respond to mental health-related presentations. Of these models, many are a secondary response service, meaning that emergency services will already be on scene, and will refer to the co-response model following initial assessment.

An evaluation of the co-response ‘Street Triage’ model in Birmingham and Solihull National Trust UK found a reduction in the number of involuntary detentions invoked, and the cost of the Street Triage model was offset by these reductions [43].

A further evaluation of the West Midlands ‘Mental Health Triage’ model identified several benefits including improved patient outcomes, a positive cultural shift in the way emergency services view mental health crises, improved service collaboration, and more collaborative information sharing [43].

In 2016, an evaluation of nine sites in England was undertaken and assessed the impact of street triage on involuntary detentions [44]. The evaluation found a significant reduction in involuntary detentions, consistent with the findings from other studies [44].

A descriptive study of the “Psychiatric Emergency Response Team” (PAM) paramedic and clinician co-response service in Stockholm County, Sweden, was undertaken over a 12-month period [45]. Data were extracted from medical and police records and demonstrated that one third of patients’ assessment did not require any further action after psychiatric assessment [45].

In an Australian evaluation of the Northern Police and Clinician Emergency Response (NPACER) co-response police and clinician model, trend analysis and cross-comparison found that NPACER reduced involuntary detentions by 86% when compared with the single police-only response [6]. The authors concluded that NPACER facilitated least restrictive care and hospital diversion, as well as freed up police resources [6].

Police and clinician co-responder models and their effectiveness were examined in a systematic review in 2018 [46]. Twenty-six studies were included, and patient characteristics and involuntary detention rates were examined [46]. Though noting some possible reduction in the use of involuntary detentions, the authors provided no definitive conclusions as to the effectiveness of the co-response models and recommended further research into the topic [46].

Further qualitative research examining police officers’ attitudes to responding to mental health crises demonstrated that police often invoke involuntary detention in the absence of other mechanisms to support the person in the community, and they felt that mental health co-responder models provided a good alternative to detention and transport to an ED [47].

A further literature review did not produce any research into the tri-response PACER model’s effectiveness or association with involuntary detentions. Whilst there are some studies examining the police/clinician or ambulance/clinician co-response models, tri-response models have not yet been evaluated and may yield different results. The PACER model in the ACT is adopted from the tri-response Birmingham UK model and has the capacity to operate as a first responder team.

### 1.9. Objectives

**Figure 1:**
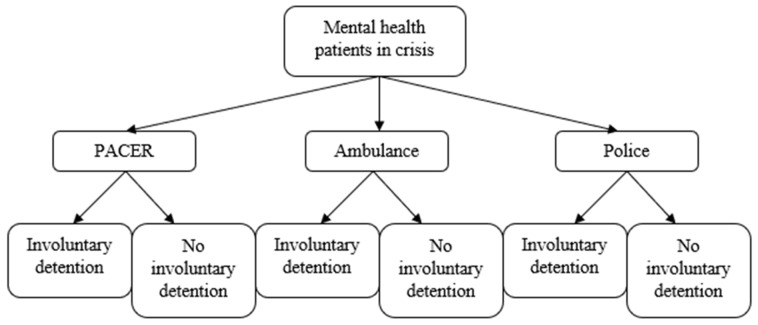
Primary outcome.

**Figure 2:**
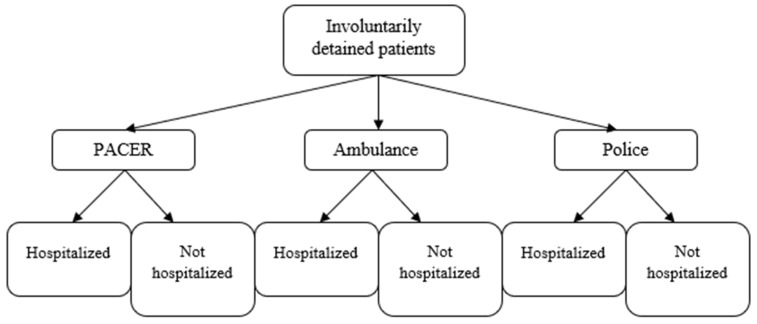
Secondary outcome.

The aim of this study is to determine the association between three approaches to responding to an acute mental health crisis (PACER, police, or ambulance as the cohorts) and rates of involuntary detentions, and to estimate the strength of that association. Objective one is described in Figure 1. Additionally, we aim to determine the association between the cohorts and rates of post-involuntary-detention hospitalization, and to estimate the strength of that association. This objective is described in Figure 2. We further aim to determine the probability of being involuntarily detained and being diverted from hospital in the PACER cohort, and to estimate the strength of that association. Finally, we aim to describe the patient characteristics and outcome of patients assessed by PACER during the study period.

### 1.10. Hypothesis

Rates of involuntary detention will be lower in the PACER cohort than the police and ambulance cohorts, and PACER rates of post-detention hospitalization will be higher.

## 2. Materials and Methods

### 2.1. Study Design

This is a cohort study using a retrospective design over a 12-month period using records from December 2019 to December 2020.

Retrospective cohort studies allow researchers to formulate ideas about the associations and potential relationships between the independent variable (e.g., PACER) and dependent variables (involuntary detentions, hospital diversions) without determining causal effect [48,49,50,51].

There are several strengths to retrospective cohort studies. These include large sample sizes and statistical power based on the availability of large databases of data which have already been collected [48,49,50,51]. Furthermore, both prospective and retrospective cohort studies have high accuracy and efficacy as their main advantages [48,49,50,51]. A primary feature of observational studies is the identification of participants and their exposure to a risk factor, intervention, or outcome, and this is assessed at the study’s starting point [49].

This study will use PACER as the exposure, and police and ambulance cohorts as comparison.

### 2.2. Setting

The study pertains to a single site in the Canberra Region, Australian Capital Territory (ACT).

Canberra Health Services provides a comprehensive range of governmental primary, secondary, and tertiary public health services to the ACT region. Mental Health, Justice Health and Alcohol and Drug Services (MHJHADS) is a division within Canberra Health Services and includes inpatient mental health and assessment units, crisis assessment and management teams, community mental health and outpatient psychiatry teams, rehabilitation services, and step up/step down facilities across all age groups.

Under the Mental Health Act 2015, all patients subject to involuntary detention must be brought to the Canberra Hospital Emergency Department as an approved facility for involuntary assessment [27]. The Canberra Hospital is the largest public hospital in the ACT region, providing a range of services including 672 inpatient beds, ambulatory and outpatient services, and pathology.

### 2.3. Participants

The participants included in this study are those who have received either a police, ambulance, or PACER response for a mental health crisis. For the purpose of this study, participants are those who are experiencing a mental health crisis where 000 police or ambulance emergency assistance has been requested. Generally, calls to emergency services demonstrate an immediate need for response and may include suicidal behavior, self-harm, or psychotic illness.

### 2.4. Inclusion Criteria

The sample will include all PACER, police, and ambulance responses to mental health crises between December 2019 and December 2020.

### 2.5. Exclusion Criteria

Occasionally, PACER will be diverted to prioritize time-critical jobs that are not related to mental health patients, such as cardiac arrest, if they are the closest emergency vehicle within range of the incident. These incidents will be excluded as they do not relate to the study.

### 2.6. Dispatching Protocol

The ACT Policing Communications Sergeant is responsible for dispatching PACER through the 000-emergency system. This includes communicating with the ACT Ambulance Service and mental health services using a triaging system to determine which agency should respond in the first instance. The protocol is confidential, but is dependent on availability, location, and the likelihood of requiring a secondary team.

### 2.7. Ethical Considerations

Permission to conduct this retrospective cohort study was granted by the Canberra Hospital Service Clinical Ethics Committee and the La Trobe University Human Research Ethics Committee prior to conducting the evaluation.

The ethical consideration for this study is that of patient consent to participate in the study. We sought and were approved for a waiver of consent for use of patient records as per Section 2.3.10 of the National Statement on Ethical Conduct in Human Research. The request for a waiver of consent was based on the following:

- Section 2.3.10(a): This research carries no more than low risk as the project will be at a cohort level, and not an individual level.
- Section 2.3.10(c): It is impractical to obtain consent from patients, as they are subjected to mental health orders and/or guardianship orders. Seeking consent may cause more distress for the person/s responsible than not seeking consent.
- Section 2.3.10(e and f): There is sufficient protection of their privacy, as individual identifiers will not be collected as part of the project.
- Section 2.3.10(g): Currently, there is a high rate of unnecessary involuntary detention of people living with mental illness in the ACT. The outcomes of this evaluation will provide evidence for the improvement in service delivery for groups of very marginalized and vulnerable people, through providing least restrictive and patient-centered options for assessment, treatment, and care. The request for a waiver of consent is considered justified as the benefits of the evaluation greatly outweigh any potential risks.

## 3. Variables/Outcomes

### 3.1. Primary Outcomes

The primary study outcome is the rate of involuntary detention in the three cohorts. Involuntary detentions will be compared with the total incident number to determine the rate of detention by cohort (PACER, police, ambulance).

### 3.2. Secondary Outcomes

Post-detention hospitalization will be explored using involuntary detention data. Post-detention hospitalization is the process whereby a patient who is subject to involuntary detention is then hospitalized in a psychiatric unit for that episode of care. It is considered that hospitalization justifies the use of involuntary detention, as the patient’s mental illness was acute and requiring emergency assessment at the time that the involuntary detention was invoked.

Additionally, this study will review hospital diversion within the PACER model. Hospital diversion is defined as a process whereby a patient is assessed, and a treatment plan established in the community, negating the need for transfer to hospital. Hospital diversion data will be extracted from the PACER data collection form. For every PACER job, the police officer and paramedic will be asked if they would have invoked an involuntary detention if they were working outside of PACER in their general duties. This data will provide further evidence about the association between PACER and involuntary detention.

Finally, the study will describe the characteristics of patients assessed by PACER including formulation, diagnosis, outcome, and general grouped demographics such as utilization by age.

## 4. Data Sources

### 4.1. Involuntary Detention and Hospitalization Data

Copies of involuntary detention forms and secondary statistics kept by the Office of the Chief Psychiatrist will be used to extract data relating to the number of involuntary detentions invoked by PACER, police, and ambulance. The data will be used to measure the rate of involuntary detentions, post-detention hospitalization, and hospital diversions for each cohort. Post-detention hospitalization will be calculated using involuntary detention data from the Mental Health Tribunal Liaison and Mental Health, Alcohol and Drug Service, Justice Health, Integrated Care Record (MAJICeR) databases.

### 4.2. PACER Data Extraction Form

The PACER data extraction form is completed collaboratively by the PACER team for each presentation and is stored in the CHS electronic record. These records will be used to extract PACER case mix and outcome data.

## 5. Bias

As this is a retrospective cohort study, participant drop out or “loss to follow up” is not a factor which would potentially cause bias. However, the retrospective approach can increase the risk of bias through the greater likelihood of missing data [48,49,50,51]. It is considered that any missing data would occur at random (missing at random). Missing data is a common issue within cohort and observational studies both within prospective and retrospective designs, and can lead to loss of power, and wider confidence intervals due to a reduced sample size [48,49,50,51]. We have developed a plan for dealing with missing data which includes the following:

- Reducing the amount of missing data through mandatory documentation policies;
- Cross-referencing of PACER data forms by researchers, mental health clinicians, and police for finalization and closure;
- Calculating the sample size needed to measure effect;
- Recovering the missing data through contacting the author of the PACER forms to request completion;
- Further investigation of the clinical files by the researchers, depending on the missing variable;
- Calculating randomness using Little’s MCAR test.

We consider that there is a potential for missing data across different datasets, however, this is likely to be in small numbers based on the strict documentation protocols within the three agencies. However, this may be a single missing variable within the PACER data form, or a PACER data form completely missing.

If the listed strategies do not resolve the missing data, we will undertake single or multiple imputation depending on what data are missing. Single imputation refers to the missing variable being replaced with a single value that would best represent the mechanism of the missing data [52,53]. Multiple imputation is a general approach that is easily accessible using STATA and is a flexible and practical approach for managing missing data [52,53]. Using this approach, missing values are assigned using the predictive distribution of data that has been observed multiple times.

Mental health incident data from police and ambulance will be provided only as total number of jobs for the study period. Whilst there is a possibility that jobs may have been coded incorrectly, this is unlikely to be a significant source of bias, although it cannot be adjusted for, as missing data cannot be identified due to the reporting systems of each database.

We consider that participants in the two police and ambulance cohorts are representative of the participants in the PACER cohort, as they meet the same inclusion criteria, minimizing the risk of selection bias.

Confounding is common in observational studies and refers to an unknown variable which distorts the effect being measured, leading to an under- or over-representation of the association between the variables [48,49,50,51]. To keep the study population consistent, we will remove all data where PACER was redirected to a medical emergency. Furthermore, we will not include non-face-to-face PACER jobs, such as phone assessments, in the analysis of PACER involuntary detentions and hospital diversions.

A likely confounder is the risk of hospitalization by diagnosis. For example, a person experiencing a situational crisis or emotional dysregulation may be less likely to be involuntarily detained than someone who is acutely suicidal or psychotic. We will use multivariate regression analysis to build a regression model for the outcome and exposure, as well as the various diagnostic variables. The effect of the variable of interest (involuntary detention, hospital diversion) can be examined with confounding variables held constant. This will only be possible for the PACER data, as police and ambulance data provide only job numbers within the study period.

Repeat participants can represent a challenge in observational research as repeated presentations can skew data in a particular direction. We will screen for this during the data extraction phase with consideration to omitting participants whose repeated presentations may cause bias in the data analysis.

## 6. Sample Size

Sample size is an important factor in determining the validity of the study in terms of the statistical power necessary to reject or accept a hypothesis. Correlation observational studies such as cohort studies use a null hypothesis (PACER does not reduce involuntary detentions), which is set to be rejected with an alternative hypothesis (PACER does reduce involuntary detentions) [48,49,50,51].

Effect size refers to the difference expected in the study outcome as a result of the intervention being measured and refers to the magnitude of the relationship between the two variables [54,55,56]. We will undertake a study size calculation using a confidence interval of >95%, population size, and a 5% margin of error.

Despite extensive review of the literature, we were unable to obtain any studies relating to the tri-service PACER model which explored rates of involuntary detentions. Instead, we referred to a trend analysis and cross-comparison study of the police and clinician co-response NPACER model in Victoria, Australia, to determine the association of NPACER and involuntary detentions [6]. Within a six-month period, the rate of involuntary detention by NPACER was 16%, compared with 100% during times when NPACER was not operating [6]. Using a g-power sample size calculator with a proportion of 0.16 and confidence level of 95%, the estimated sample size required to demonstrate an association between PACER and involuntary detentions is 7. Formal data extraction will be completed as part of the formal study, however, an initial review indicates that approximately 1200 records will be included.

## 7. Statistical Methods

We will use the STATA statistical software, version 18.0 (StataCorp, College Station, TX, USA) package for data management and analysis.

We will use relative risk (RR) calculation to determine the risk of being involuntarily detained in the PACER, police, and ambulance cohorts. Relative risk refers to the probability of an event occurring in the exposed group versus the probability of the event occurring in the non-exposed group, and we will calculate this using standard calculations [57,58].

Following calculation of the RR, we will determine the confidence interval (CI) to measure the degree of certainty of the study sample using standard CI calculations. A 95% confidence interval will contain the true value of the risk ratio 95% of the time and represents a high degree of certainty [59]. We will apply this statistical analysis to examine the relationship between PACER and hospital diversion.

We will use logistic regression using univariate and multiple analysis including gender, age, and diagnostic formulation as independent variables and involuntary detention as the dependent variable. We will use multiple analysis to control for potential confounding amongst the variables. Using this method, we will estimate the probability of being involuntarily detained or diverted from hospital by variable, expressed as the odds ratio (OR). Whilst there is no comparator for hospital diversions, these data will provide further understanding of the capacity of PACER in meeting its initial targets of linking patients with community support or de-escalating a social crisis, and accurately identifying those patients who do need to go to hospital for treatment.

We selected RR in our analysis of rates of involuntary detention across the cohorts, as this represents the probability of occurrence of an event or outcome. Given that we are using PACER as the exposure group and police and ambulance as the non-exposed group to examine involuntary detentions as the outcome, we consider RR to be the most appropriate analysis to answer our research question.

Alternatively, we selected OR for the logistic analysis, as this also indicates the nature of the association between the exposure and is a common calculation method for multivariate analysis technique.

Finally, we will describe the patient characteristics and outcome of patients assessed by PACER, using total and percentage.

## 8. Data Management

The primary risk in this study relates to data security and integrity, as well as the deidentification of data. We have developed a strict management plan for the storage, deidentification, re-coding, disposal, and checking of data, which is consistent with national and local legislation and hospital policies and procedures.

### 8.1. Data Storage

Extracted data will be stored in the CHS (Canberra Health Services) information technology system. The system has high levels of protection as per the Commonwealth *Privacy Act* 1988 [60] and the *Health Records (Privacy and Access) Act* 1997 (ACT) [61] including password protection and restricted access to business folders.

### 8.2. Procedure for Deidentifying Data

During the extraction phase of the study, we will not extract identifying patient data such as name, date of birth, address, or any other identifying data. We will not record a patient’s age; rather, we will code into three age groups, adding an extra layer of security.

### 8.3. Data Disposal

At the end of the study, the research database will be archived within an allocated business folder of the CHS database. We will follow the CHS procedures and national and local legislation.

At the end of the study period, archived data will be destroyed after seven years via the CHS Clinical Records Service, following standard data disposal processes defined in the policy.

### 8.4. Data Checking

Error in data entry may occur when PACER clinicians complete the PACER data extraction form. Checks will be undertaken to identify unlikely values such as ages outside of 8–90 years.

We will cross-reference the PACER data extraction form with the various electronic records to reduce systematic errors. The form will be cross-referenced by the police officer before sending it to the PACER project team.

It is unlikely that duplication of data will occur, given the high level of documentation requirements for each patient’s job.

### 8.5. Data Re-Coding

Data extracted will be re-coded into new variables and applied to a code book.

## 9. Results

We will present our findings using the STROBE checklist for reporting cohort studies. We will provide total participant numbers for the PACER, police, and ambulance cohorts and RR calculations for both involuntary detention and diversion from hospital. We will provide standard logistic regression tables for involuntary detentions and diversion from hospital for the three cohorts. We will present characteristic data of PACER presentations, including age and gender demographics, reason for PACER presentation, formulation, and outcome, displayed in tables using total distribution and percentage.

We will provide a comprehensive discussion of the study findings and their interpretation and describe the limitations of the study and opportunities for future research.

Identifying patient data will not be included in the evaluation results.

### Limitations

Whilst observational studies are an effective and robust method of establishing association, some limitations exist. The retrospective approach increases the likelihood of bias in the cohort or experimental group as the risk of missing data is higher [50,51,52,53]. Retrospective studies may be further weakened due to the dataset available not being designed for the study; rather, researchers make use of what data are already available, which may limit the overall quality of the study [48,49,50,51].

Presentation is a potential confounder for this study, as highly agitated or violent patients may be more likely to get a full police response than a PACER response, which has only a single police officer, and a person living with mental illness suffering severe injuries is more likely to receive an ambulance response.

The study cannot adjust for individual decision making of PACER staff. Whilst all PACER staff were recruited against high standards of crisis experience, it is possible that some clinicians will be more risk averse than others.

Imputation requires the researcher to carefully consider the plausibility of the causes of the missing data [52,53]. Single imputation may not adequately account for uncertainty within results, and multiple imputation is time-consuming and requires a higher level of researcher knowledge [52,53].

The involuntary detention data from the police and ambulance data have not been tested for validity or reliability and may be subject to misclassification bias due to incorrect categorization within the relevant reporting systems. Because data systems within the police and ambulance service are different, we are unable to distinguish between non-differential and differential misclassification, should it occur.

## 10. Conclusions

This study will provide evidence of the effectiveness of the PACER tri-response model through measuring the association between the model and the rate of involuntary detentions. To date, very limited data exist that explore the impact of the tri-response model of PACER, particularly its capacity to reduce involuntary detentions and emergency department presentations. This study provides evidence for the value of inserting mental health clinicians into the emergency services space and the capacity for upskilling and changing the culture of emergency service personnel when working with people living with mental illness. The results of this study will inform health services and policy makers in guiding decisions relating to mental health crisis interventions relating to emergency services.

## Public Involvement Statement

A copy of the protocol was provided to local mental health consumer and carer groups for their awareness.

## Guidelines and Standards Statement

We are reporting a protocol for an observational study. STROBE guidelines informed how we drafted the protocol [62].
